# Does prior administration of rtPA influence acute ischemic stroke clot composition? Findings from the analysis of clots retrieved with mechanical thrombectomy from the RESTORE registry

**DOI:** 10.1007/s00415-021-10758-5

**Published:** 2021-08-20

**Authors:** Rosanna Rossi, Sara Molina, Oana Madalina Mereuta, Andrew Douglas, Seán Fitzgerald, Ciara Tierney, Abhay Pandit, Paul Brennan, Sarah Power, Alan O’Hare, Michael Gilvarry, Ray McCarthy, Georgios Magoufis, Georgios Tsivgoulis, András Nagy, Ágnes Vadász, Katarina Jood, Petra Redfors, Annika Nordanstig, Erik Ceder, Dennis Dunker, Jeanette Carlqvist, Klearchos Psychogios, István Szikora, Turgut Tatlisumak, Alexandros Rentzos, John Thornton, Karen M. Doyle

**Affiliations:** 1 Department of Physiology and Galway Neuroscience Centre, School of Medicine, National University of Ireland Galway, University Road, Galway, Ireland; 2 CÚRAM–SFI Research Centre in Medical Devices, National University of Ireland Galway, Galway, Ireland; 3 Department of Radiology, Royal College of Surgeons in Ireland, Beaumont Hospital, Dublin, Ireland; 4 Cerenovus, Galway, Ireland; 5 Metropolitan Hospital, Stroke Unit, Piraeus, Greece; 6 Second Department of Neurology, National and Kapodistrian University of Athens, “Attikon” University Hospital, Athens, Greece; 7 Department of Neurointerventions, National Institute of Clinical Neurosciences, Budapest, Hungary; 8 Department of Neurology, Sahlgrenska University Hospital, Gothenburg, Sweden; 9 Department of Interventional and Diagnostic Neuroradiology, Sahlgrenska University Hospital, University of Gothenburg, Gothenburg, Sweden; 10 Department of Clinical Neuroscience, Institute of Neuroscience and Physiology, Sahlgrenska Academy at University of Gothenburg, Gothenburg, Sweden

**Keywords:** Stroke, Mechanical thrombectomy, Bridging-therapy, Thrombus histology, Thrombus size

## Abstract

**Background and purpose:**

There is still much debate whether bridging-therapy [intravenous thrombolysis (IVT) prior to mechanical thrombectomy (MT)] might be beneficial compared to MT alone. We investigated the effect of IVT on size and histological composition of the clots retrieved from patients undergoing bridging-therapy or MT alone.

**Methods:**

We collected mechanically extracted thrombi from 1000 acute ischemic stroke (AIS) patients included in RESTORE registry. Patients were grouped according to the administration (or not) of IVT before thrombectomy. Gross photos of each clot were taken and Extracted Clot Area (ECA) was measured using ImageJ software. Martius Scarlett Blue stain was used to characterize the main histological clot components [red blood cells (RBCs), fibrin (FIB), platelets/other (PTL)] and Orbit Image Analysis was used for quantification. Additionally, we calculated the area of each main component by multiplying the component percent by ECA. Chi-squared and Kruskal–Wallis tests were used for statistical analysis.

**Results:**

451 patients (45%) were treated with bridging-therapy while 549 (55%) underwent MT alone. When considering only percent histological composition, we did not find any difference in RBC% (*P* = 0.895), FIB% (*P* = 0.458) and PTL% (*P* = 0.905). However, bridging-therapy clots were significantly smaller than MT-alone clots [32.7 (14.8–64.9) versus 36.8 (20.1–79.8) mm^2^, *N* = 1000, H1 = 7.679, *P* = 0.006*]. A further analysis expressing components per clot area showed that clots retrieved from bridging-therapy cases contained less RBCs [13.25 (4.29–32.06) versus 14.97 (4.93–39.80) mm^2^, H1 = 3.637, *P* = 0.056] and significantly less fibrin [9.10 (4.62–17.98) versus 10.54 (5.57–22.48) mm^2^, H1 = 7.920, *P* = 0.005*] and platelets/other [5.04 (2.26–11.32) versus 6.54 (2.94–13.79) mm^2^, H1 = 9.380, *P* = 0.002*] than MT-alone clots.

**Conclusions:**

Our results suggest that previous IVT administration significantly reduces thrombus size, proportionally releasing all the main histological components.

## Introduction

Current evidence-based treatments for acute ischemic stroke (AIS) in Large Vessel Occlusion (LVO) include both intravenous thrombolysis (IVT) and mechanical thrombectomy (MT) [1–4]. Current guidelines recommend proceeding with both treatments as soon as possible. Patients may be unsuitable for IVT but still suitable for MT. While the effectiveness of MT compared to IVT alone has been extensively demonstrated [5–10], conflicting results have been reported when comparing bridging-therapy to MT thrombectomy alone [11–15]. Questions remain as to whether pre-treatment with intravenous thrombolytics significantly affects MT success and patient outcome. There are several ongoing randomized controlled clinical trials addressing this issue.

Investigation of the properties of extracted thrombi can also help in this debate, since thrombus properties like size and composition could be influenced by IVT and have clinical significance. In this regard, we recently reported that clots extracted from patients undergoing bridging-therapy are significantly smaller compared to those of patients undergoing MT alone, although there was no difference in final recanalization outcome [16]. Furthermore, several studies have reported the wide heterogeneity in thrombus composition [17–19], which is likely to be influenced by several factors, including etiology and perhaps also thrombolysis [20].

In this study, we investigated the effect of previous IVT administration in clots removed by MT on histological composition of thrombi retrieved from a larger cohort of 1000 AIS patients, both in terms of percentage composition and also as a function of clot size.

## Materials and methods

### Patient cohort

We collected clots from 1000 AIS patients as part of the RESTORE registry of acute ischemic stroke clots. The RESTORE Registry is a registry of thrombotic material extracted via mechanical thrombectomy from patients suffering from AIS and it accounts for clots collected during the period February 2018 to December 2019 from four stroke centres in Europe. A main aim of the Registry is to analyse clots from AIS patients and correlate clot characteristics with clinical and procedural information. For this reason, patients for which no clot could be extracted are not part of the Registry and we have no information about these patients. This multi-centre prospective study was conducted in accordance with the ethical standards of the Declaration of Helsinki and its amendments [21], by approval of the regional hospital ethics committees and National University of Ireland Galway research ethics committees (16-SEPT-08).

We included only patients > 18 years, having been treated with mechanical thrombectomy for AIS and whose thrombus material was available for analysis. For each patient an anonymized data abstraction form was collected, which contained pertinent procedural data, including if IVT was administered, occlusion location, type of device used for MT, number of passes for clot removal, suspected etiology and final modified Treatment In Cerebral Ischemia (mTICI) score [22]. Grade of reperfusion after endovascular procedure was assessed by anteroposterior/lateral Digital Subtraction Angiography and certified by at least two radiologists at the hospital site. Suspected stroke etiology was reported according to the TOAST classification system [23].

### Measurement of extracted thrombus size: Extracted Clot Area

After the endovascular treatment, extracted thrombi were collected *per pass* and shipped in 10% formalin to NUI Galway, where a gross photo of each was taken with a Canon EOS 1300D Camera. Extracted Clot Area (ECA) was used as an estimate of the size of extracted clot and was measured as previously reported [17, 24, 25]. In brief, to measure the area of a fragment, the gross photo was opened with ImageJ software, the scale was set and the Polygon tool was used to draw a region of interest around the fragment. For passes involving multiple fragments we measured and summed the area of each fragment to obtain the ECA of each pass. Then, for cases involving multiple passes we summed the ECA of each pass to obtain the overall ECA.

### Histological component analysis

After having been photographed, thrombi were placed in histological cassettes for tissue processing and were paraffin embedded. 3 µm sections were cut with a microtome. Martius Scarlett Blue (MSB) staining was performed to identify the standard clot tissue components: red blood cells (RBCs), white blood cells (WBCs), fibrin (FIB), platelets/other (PTL) and non-typical thrombus components such as collagen and calcification [26]. We used machine-learning Orbit image analysis (www.Orbit.bio) for quantification purposes [27, 28]. Briefly, we created exclusion and inclusion models to distinguish regions to be excluded (e.g. background and artefact) and regions containing the tissue components of interest, enabling quantitative assessment of the proportion of each component within each clot.

In addition, we calculated the area occupied by each main component (RBC, FIB, PTL) by multiplying the component percent by the relevant ECA. For cases involving multiple passes, we summed the values of ECAxComponent for all passes.

### Statistical analysis

IBM SPSS-25 software was used for statistical analysis. Kolmogorov–Smirnov test and Shapiro–Wilk test indicated that quantitative variables did not follow a standard normal distribution. Therefore, the non-parametric chi-squared and Kruskal–Wallis tests were used to assess statistically significant difference among the groups, with a level of statistical significance set at *p* < 0.05 (two-sided). Results are reported as median [IQ1-IQ3] or number and % of cases.

## Results

### Baseline characteristics of the patients

Among the 1000 cases considered, 451 patients (45%) were treated with bridging-therapy while 549 (55%) were treated with mechanical thrombectomy alone. The total number of passes performed was 2365, with at least some clot material extracted in 1496 passes (63%), while 869 attempts were performed with no clot extraction (37%). Baseline clinical characteristics of both groups of patients are reported in Table 1. There was no significant difference between the two populations in terms of suspected etiology (*X*^*2*^ = 3.771, *P* = 0.438), first-line approach used (*X*^*2*^ = 2.417, *P* = 0.120) or revascularisation outcome (H1 = 1.189, *P* = 0.275); Table 1.

**Table 1:** Baseline clinical characteristics of the overall cohort of patients and divided by the two groups of patients, bridging-therapy and mechanical thrombectomy alone

Suspected etiology*	Overall cohort of patients (*N* = 1000)	Bridging-therapy cases (*N* = 451)	Mechanical thrombectomy-alone cases (*N* = 549)
Patients with cardioembolic suspected etiology	346 (34.6%)	152 (33.7%)	194 (35.3%)
Patients with large artery atherosclerosis suspected etiology	221 (22.1%)	111 (24.6%)	110 (20.0%)
Patients with other suspected etiology^a^	55 (5.5%)	22 (4.9%)	33 (6.0%)
Patients with cryptogenic etiology	255 (25.5%)	115 (25.4%)	140 (25.5%)
First line approach^b^			
Aspiration	657 (65.7%)	285 (63.2%)	372 (67.8%)
Stentriever	342 (34.2%)	166 (36.8%)	176 (32.1%)
Median number of attempts performed during the endovascular procedure	2 (1–3)	2 (1–3)	2 (1–3)
Final mTICI score^c^			
mTICI 0	17 (1.7%)	6 (1.3%)	11 (2.0%)
mTICI 1	12 (1.2%)	6 (1.3%)	6 (1.1%)
mTICI 2a	62 (6.3%)	30 (6.7%)	32 (6.0%)
mTICI 2b	257 (26.1%)	106 (23.7%)	151 (28.1%)
mTICI 2c	207 (21.0%)	97 (21.7)	110 (20.5%)
mTICI 3	429 (43.6%)	202 (45.2%)	227 (42.3%)

Data given as *N *(%) of cases or median (IQ1, IQ3)

^a^Other suspected etiology included: arterial dissection, pulmonary embolism, hypercoagulable states, or hematologic disorders

^b^In 1 case a gooseneck snare has been used as first-line approach to remove a calcified clot

^c^In 16 cases involving posterior circulation occlusion, it was not possible to assess final recanalization outcome (4 cases for bridging-therapy and 12 for MT alone)

*Suspected etiology was not recorded for 123 patients (12.3%), 51 of which were treated with bridging-therapy (11.3%) and 72 with MT alone (13.11%)

### Occluded vessel location

Occluded vessel location was defined by an expert radiologist at the hospital site at the beginning of procedure. In the majority of the cases, (881, 88%) the ischemic territory was located in the anterior circulation. In 100 cases (10%), the occlusion was located in the posterior circulation, while in 17 cases (2%) both anterior and posterior territories were involved. Occlusion involved a single vessel in 78% of cases (782 single occlusions), while in 216 cases (22%), two or more vessels were occluded at the same time. Specific vessels occluded, for the overall population, and according previous IVT administration, are reported in Table 2. There was no significant difference in occlusion type observed between the bridging-therapy and mechanical thrombectomy-alone groups (anterior/posterior occlusion: *X*^*2*^ = 4.575, *P* = 0.102; singular/multiple occlusion: *X*^*2*^ = 0.061, *P* = 0.804).

**Table 2:** Occluded vessels in the whole cohort of patients and in the two groups, bridging-therapy and mechanical thrombectomy alone

Occlusion type	Occluded vessel(s)	All the cases (*N* = 1000)	Bridging-therapy cases (*N* = 451)	Mechanical thrombectomy-alone cases (*N* = 549)
Singular occlusion (*N* = 781)	MCA,M1	424 (42.4%)	202 (44.9%)	222 (40.4%)
	MCA,M2	124 (12.5%)	53 (11.8%)	71 (13.0%)
	MCA,M3	5 (0.5%)	4 (0.9%)	1 (0.2%)
	MCA not specified	10 (1.0%)	8 (1.8%)	2 (0.4%)
	Vertebro/basilar	78 (7.8%)	26 (5.8%)	52 (9.5%)
	ICA and ICA terminus	131 (13.1%)	55 (12.2%)	76 (13.9%)
	PCA	8 (0.8%)	2 (0.4%)	6 (1.1%)
	ACA	2 (0.2%)	1 (0.2%)	1 (0.2%)
Multiple occlusion (*N* = 216)	Tandem occlusions	109 (11%)	52 (11.6%)	57 (10.4%)
	Other dual occlusions	41 (4.1%)	19 (4.2%)	22 (4.0%)
	MCA, multiple segments/branches	27 (2.7%)	12 (2.7%)	15 (2.7%)
	Three or more occlusions	39 (3.9%)	16 (3.6%)	23 (4.2%)

Data given as *N *(%) of cases

*MCA* middle cerebral artery, *ICA* internal carotid artery, *PCA* posterior cerebral artery, *ACA* anterior cerebral artery, *CCA* common carotid artery

### Analysis of Extracted Clot Area

**Fig. 1:**
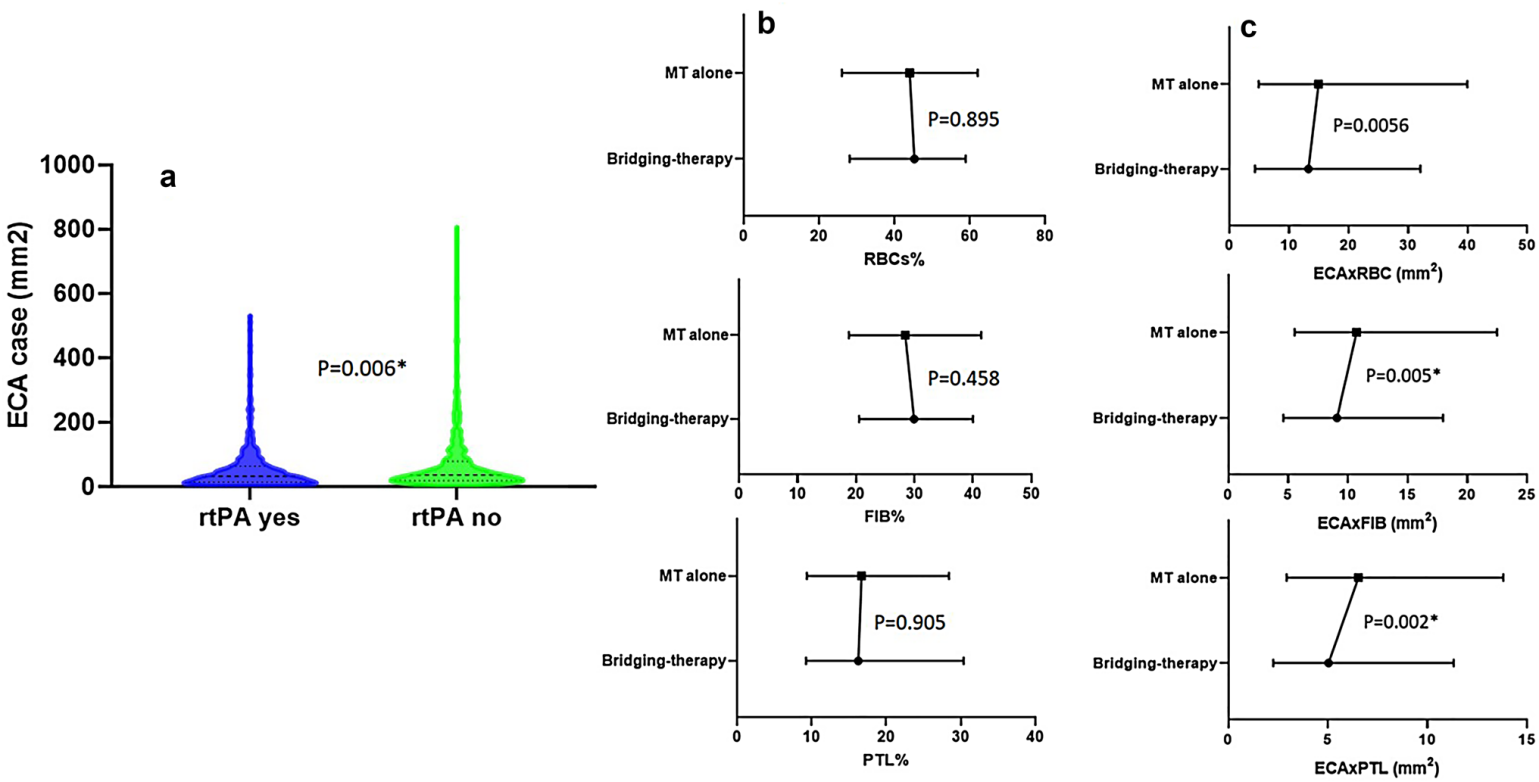
Violin plot depicting variation and difference in Extracted Clot Area (**a**); Median values with IQ[1–3] range depicting difference in percentage of main histological components (**b**) and the area occupied by each main component, (expressed as ECA multiplied by component fraction) (**c**), between clots retrieved from patients undergoing bridging-therapy or mechanical thrombectomy alone

Analysis of size of extracted clot (expressed as Extracted Clot Area) showed that clots from patients undergoing bridging-therapy were statistically significantly smaller than clots retrieved from patients treated with MT alone, (Fig. 1a; median ECA was, respectively, 32.7 [14.8–64.9] versus 36.8 [20.1–79.8] mm^2^ *N* = 1000, H1 = 7.679, *P* = 0.006*).

Bridging-therapy clots were also associated with a significantly lower number of fragments compared to MT alone (2 [1–4] versus 3 [2–5], *N* = 1000, H1 = 4.058, *P* = 0.044*).

### Histological components analysis

Histological component analysis revealed no difference in main histological components between bridging-therapy and MT-only clots (Fig. 1b). Median RBCs composition was 45 [28–59]% for bridging-therapy and 44 [26–62]% for MT-alone clots (H1 = 0.018, *P* = 0.895); median FIB composition was 30 [21–40]% for bridging-therapy and 29 [19–41]% for MT alone (H1 = 0.552, *P* = 0.458); median PTL composition was 16 [9–30]% for bridging-therapy and 17 [9–28]% for MT alone (H1 = 0.014, *P* = 0.905).

Further analysis was then performed combining size and histological composition (Fig. 1c). The amount of RBC was lower in bridging-therapy compared to MT-only clots, although not statistically significant (median ECAxRBC was 13.25 [4.29–32.06] mm^2^ versus 14.97 [4.93–39.80] mm^2^, H1 = 3.637, *P* = 0.056). The amount of FIB and PTL was statistically significantly lower in bridging-therapy clots compared to MT-only clots (respectively, median ECA × FIB was 9.10 [4.62–17.98] mm^2^ versus 10.54 [5.57–22.48] mm^2^, H1 = 7.920, *P* = 0.005* and median ECA × PTL was 5.04 [2.26–11.32] mm^2^ versus 6.54 [2.94–13.79] mm^2^, H1 = 9.380, *P* = 0.002*), Fig. 1c.

## Discussion

There are several studies in the literature comparing final recanalization rate and other clinical outcomes for patients treated with bridging-therapy or MT alone, although there is not yet a clear consensus of opinion [11–16]. Nevertheless, bridging-therapy, i.e. the combination of IVT and mechanical thrombectomy, is currently the standard of care for large vessel occlusion (LVO) patients with a NIHSS-score of 6 or greater [29] provided that they fulfil tPA eligibility criteria [1–4]. Although it could be very informative, there is very little published about the influence of IVT on the characteristics of the retrieved clot, such as its general composition and structural organization. Our recent study comparing the size of extracted thrombi showed that bridging-therapy clots were significantly smaller than MT-only clots [16], a finding that is reproduced in the present larger study.

In the present study, we sought to assess if thrombolysis also altered clot composition. In this respect, we investigated percentage composition of main components and we also undertook a combined size and compositional analysis. Red blood cells were the main histological component for both groups of clots, followed by fibrin and then by platelets/other. We did not find any difference between the clots retrieved from the two groups of patients in terms of percent composition of main histological components. However, when size was taken into account, bridging-therapy clots contained less RBC (mm^2^) and statistically significantly less fibrin and platelets/other (mm^2^) compared to clots from patients treated with MT alone, directly relatable to the smaller size of bridging-therapy clots. Whilst the effect on RBC (mm^2^) did not quite reach statistical significance, the very clear trend suggests that tPA administration reduces the content of all main components proportionally.

A previous study observed that previous IVT administration was associated with prominent changes in the architecture of the fibrin components and platelet count [30] of the retrieved clots. In particular, the phenomenon was termed as “thinning”, referring to the dissociation of superficial layers of fibrin fibres, which was remarkably notable only in clots retrieved from patients treated with bridging-therapy [30]. An earlier study observed how clots retrieved after IVT treatment were more porous and with more branching points suggesting that thrombolytic therapy is associated with significant changes in the structural composition, and in particular in the fibrin network architecture of the retrieved clot [31]. Also a recent study investigating 3D organization of AIS thrombi showed how fibrin formed a grid-like or a sponge-like pattern in clots from patients pre-treated with IVT, with thinner and less densely packed fibrin fibres [32].

However, it has been also observed that sensitivity to fibrinolytic agents might depend on original thrombus composition. In this respect, RBC-rich thrombi seem to have higher sensitivity to rtPA [33, 34] probably due to their looser architecture [35], which allows for an increased penetration of thrombolytic agents into the clot [36].

Our findings show that IVT administration, even when it does not result in the complete dissolution of the occlusive thrombi, will reduce clot size. Our findings also suggest that fibrinolytic activity of IVT causes a proportional decrease of the three main histological clot components, maintaining the percentage ratio of main components within the clot. However, we acknowledge that we have no information of clot composition before mechanical thrombectomy, which would be needed for confirmation. Our findings also showed that bridging-therapy resulted in a lower number of fragments retrieved than with mechanical thrombectomy alone. However, further study is needed before any definitive conclusion can be drawn, as many factors during thrombectomy and during clot processing could impact on the number of fragments.

### Study limitations and strengths

It must be acknowledged that our study on the effect of tPA is limited to cases where mechanical thrombectomy was performed and at least part of the clot was extracted. We do not have information for patients that recanalised after IVT alone or for those that had unsuccessful thrombectomy, with no clot extracted. The two patient cohorts differ clinically regarding eligibility for tPA. MT-only patients would have been ruled ineligible for tPA treatment for reasons such as time window exclusion, risk of haemorrhage or ongoing anticoagulation for atrial fibrillation [1–4]. Also, length of time since stroke onset could influence thrombus size. Patients receiving bridging-therapy often have a shorter time window from symptoms onset to hospital admission. Further studies should address the effect of time frame on thrombus size. Furthermore, we also acknowledge the lack of complete information about eventual anticoagulation regimes of the patient, dosage and specific thrombolytic agents administered to bridging-therapy patients, although this should be addressed in future studies.

However, despite these limitations, we observed a good similarity between the two groups of patients in terms of baseline and procedural characteristics including suspected etiology, occluded vessel treated, first-line approach for endovascular procedure and final revascularisation outcome. Furthermore, the main strength of this study is the large patient population analysed, arising from four dedicated stroke centres in Europe and analysed by a specialized core laboratory, which gives robustness to our findings.

## Conclusion

The present study highlighted interesting differences between AIS thrombi retrieved from patients treated with bridging-therapy or MT alone. Bridging-therapy clots are smaller and with significantly reduced fibrin and platelet/other area (mm^2^), but with a percentage of components similar to the MT-alone clots. These findings indicate that IVT administration significantly reduces the size of the thrombus, proportionally releasing all main histological components while carrying out its fibrinolytic activity.

## Data Availability

Data may be available upon reasonable request.
